# Response surface optimization of abiotic elicitors for betalain production and antioxidant capacity in *Celosia argentea* cell suspension cultures

**DOI:** 10.1038/s41598-025-31876-4

**Published:** 2025-12-15

**Authors:** Thapagorn Sang A Roon, Poramaporn Klanrit, Poramate Klanrit, Pornthap Thanonkeo, Jirawan Apiraksakorn, Sudarat Thanonkeo, Sirinya Sitthirak, Preekamol Klanrit

**Affiliations:** 1 Graduate School, Khon Kaen University, Khon Kaen, 40002 Thailand; 2 Department of Biotechnology, Faculty of Technology, Khon Kaen University, Khon Kaen, 40002 Thailand; 3 Research Group of Chronic Inflammatory Oral Diseases and Systemic Diseases Associated with Oral Health, Department of Oral Biomedical Sciences, Faculty of Dentistry, Khon Kaen University, Khon Kaen, 40002 Thailand; 4 Department of System Biosciences and Computational Medicine, Faculty of Medicine, Khon Kaen University, Khon Kaen, 40002 Thailand; 5 Cholangiocarcinoma Research Institute, Faculty of Medicine, Khon Kaen University, Khon Kaen, 40002 Thailand; 6 Fermentation Research Center for Value Added Agricultural Products (FerVAAP), Khon Kaen University, Khon Kaen, 40002 Thailand; 7 Walai Rukhavej Botanical Research Institute (WRBRI), Mahasarakham University, Maha Sarakham, 44150 Thailand; 8 School of Allied Health Sciences, Walailak University, Nakhon Si Thammarat, 80161 Thailand

**Keywords:** Plant cell culture, Elicitor compounds, Pigment biosynthesis, Free radical scavenging, Amaranthaceae, Statistical optimization, Biochemistry, Biotechnology, Plant sciences

## Abstract

Betalains are natural pigments with diverse biological properties found *in Celosia argentea* var. *plumosa*, a member of the Amaranthaceae family. This study established an optimized cell suspension culture system for enhanced betalain production from *C. argentea* var. *plumosa* through combined elicitor treatment. Three elicitors were evaluated: 6-benzylaminopurine (BAP), methyl jasmonate (MeJA), and copper sulfate (CuSO_4_), using response surface methodology (RSM) based on central composite design (CCD). Under standard conditions, maximum total betalain content (TBC) and dry weight reached 35.61 mg/L (1.95 mg/g DW) and 19.90 g/L, respectively, on day 15. The optimal formulation consisted of 2.28 µM BAP, 49.97 µM MeJA, and 6.71 µM CuSO₄, applied during the exponential growth phase on day 9. These optimized conditions achieved a 3.9-fold increase in betalain production on day 15, reaching a maximum TBC of 139.99 mg/L (7.54 mg/g DW) with biomass of 16.90 g/L. Additionally, betalain extracts from cells cultured under optimal conditions demonstrated higher antioxidant capacity than unoptimized culture extracts. These findings suggest that using statistical experimental design with combined elicitors provides an optimized platform for scalable betalain production with enhanced bioactive properties. This information will be valuable for food, pharmaceutical, and cosmetic industries requiring natural colorants with functional benefits.

## Introduction

Betalains are water-soluble natural pigments stored in plant cell vacuoles that produce the vibrant colors observed in species from the order Caryophyllales^1^. These pigments occur across multiple plant families, such as Amaranthaceae, Cactaceae, Basellaceae, and Portulacaceae^2^. The foundation of all betalains is betalamic acid, a shared molecular structure that combines with either *cyclo*-DOPA derivatives to produce betacyanins (displaying red-violet hues) or with diverse amino acids and amines to generate betaxanthins (exhibiting yellow-orange colors). The distinct structural compositions of these compounds account for the different color profiles between the two betalain subgroups^3^. These pigments have significant potential for commercial uses, particularly as natural coloring agents in food products, beverages, and cosmetic formulations^4^. Furthermore, betalains demonstrate notable bioactive properties, such as antioxidant, anticancer, antimicrobial, and anti-inflammatory effects, positioning them as promising candidates for pharmaceutical and therapeutic applications^5^.

Due to the advantages of betalains, researchers are increasingly attempting to utilize these pigments for various applications. Red beetroot (*Beta vulgaris*) serves as the major commercial source for betanin, one of the most important and well-characterized betalain compounds^6,7^. Other significant sources of betalains include leafy amaranth (*Amaranthus tricolor*), cactus pear (*Opuntia ficus*-*indica*), dragon fruit cactus (*Hylocereus costaricensis*), quinoa (*Chenopodium quinoa*), *Bougainvillea* spp. and common cockscomb (*Celosia argentea*)^8–14^. However, obtaining betalains from natural sources presents several disadvantages, including high labor and production costs, large area requirements, climate- and geography-dependent pigment production, possible contamination risks, and inconsistent pigment yields^15,16^. Consequently, in vitro cell culture-based pigment production has emerged as a practical alternative to satisfy the expanding market demand^17^.

Numerous investigations have documented the successful production of betalains through various in vitro culture systems, including hairy root cultures, callus cultures, and cell suspension cultures^17^. The majority of betalain-production platforms have been established using *B. vulgaris*, *A. tricolor*, *O. ficus*-*indica*, *H. costaricensis*, *Stenocereus queretaroensis*, *C. quinoa*, and two varieties of *C. argentea*, i.e., *C. argentea* var. *cristata* and *C. argentea* var. *plumosa*^8–11,18–21^. These studies have confirmed the potential for controlled betalain biosynthesis under laboratory conditions. However, achieving high biomass accumulation and optimal betalain production, particularly in cell suspension culture systems, remains challenging due to the complex interactions of multiple critical parameters. Current optimization approaches typically focus on individual factors such as carbon source composition, plant growth regulator (PGR) types and concentrations, precursor molecule availability, and elicitor treatments^12,17,19,21–23^. The specific requirements and optimal concentrations of these factors exhibit considerable variation among different plant species and cell lines, creating substantial challenges for optimization.

Existing literature reveals notable limitations in current optimization methodologies, particularly regarding the systematic evaluation of synergistic effects among multiple parameters. Most previous studies have employed traditional one-factor-at-a-time experimental designs, which do not effectively demonstrate the interaction between variables, representing a significant knowledge gap in betalain production optimization. Response surface methodology (RSM) offers a powerful statistical approach for concurrently evaluating multiple variables and their interactions, enabling more efficient identification of optimal production conditions^24^. Although this methodology has been used in selected areas of plant and bioactive compound research, it remains underutilized in betalain production systems^24–27^.

In our previous studies, we isolated a betalain-producing cell line and established a cell suspension culture of *C. argentea* var. *plumosa*, determining the optimal culture conditions for betalain production and accumulation. The results demonstrated that BAP is effective for betalain production^27^. While a few studies have investigated elicitation and betalain production in *H. costaricensis*, *B. vulgaris*, and *C. argentea* var. *cristata* cell cultures, research on elicitation strategies for suspension-cultured cells (SCCs) of *C. argentea* var. *plumosa* remains limited^21,23,28^. A recent investigation identified several biotic and abiotic elicitors that promote betalain production in cell suspension cultures of this species. Among these, copper sulfate (CuSO_4_) at 6.40 µM proved to be the most effective elicitor for betalain production^29^. However, the potential of combined abiotic elicitors for enhancing betalain production has not been explored, representing a substantial opportunity for process improvement. Therefore, this study aims to address these critical gaps by applying RSM to optimize combined abiotic elicitor treatments for enhanced betalain production in *C. argentea* var. *plumosa*. The betalain production and antioxidant capacity of the extract were also evaluated.

## Materials and methods

### Plant material

*C. argentea* var. *plumosa* plants were grown in Khon Kaen Province, Thailand, from which samples were collected for experimental purposes. No specific permits were required for the collection of this non-endangered plant species. Plant identification was confirmed, and a voucher specimen was deposited at the Department of Biology, Faculty of Science, Khon Kaen University (herbarium specimen number: KKU25556). The red-callus cell line developed in this study was submitted and registered with the Intellectual Property Center, Khon Kaen University (*Celosia argentea* var. *plumosa* PrK01).

### Callus culture and maintenance

The red calli were obtained from a previous study^27^. Briefly, *C. argentea* var. *plumosa* seeds were surface sterilized by sequential treatment with 70% (v/v) ethanol for 5 min, followed by 0.6% (v/v) sodium hypochlorite for 15 min. The seeds were then carefully rinsed with distilled water to eliminate all surfactant residues. Following surface sterilization, the seeds were placed on semi-solid Murashige and Skoog (MS) medium (PhytoTech Labs, Inc., Lenexa, KS, USA) containing 3% (w/v) sucrose and 0.6% (w/v) of the gelling agent Gelzan™ (PhytoTech Labs, Inc., Lenexa, KS, USA). The culture medium pH was adjusted to 5.8 prior to autoclaving.

For callus induction, 30-day-old red hypocotyls of *C. argentea* var. *plumosa* (1 cm in length) were excised and cultured on optimized callus induction medium (CIM) comprising MS salts and vitamins supplemented with 3% (w/v) sucrose, 0.2% (w/v) Gelzan™, 1 mg/L 2,4-dichlorophenoxyacetic acid (2,4-D), and 0.1 mg/L 6-benzylaminopurine (BAP) at pH 5.8. Cultures were maintained at 25 ± 2 °C under 45 µmol m⁻² s⁻¹ light intensity with a 16 h photoperiod. After four weeks, red-colored semi-friable callus cells exhibiting rapid growth characteristics were selected and subcultured every two weeks on the same CIM formulation under identical culture conditions. Through successive subculturing (48 cycles), completely friable red cell lines with optimal growth rates were established.

The established cells were maintained in MS medium supplemented with 3% (w/v) sucrose, 0.2% (w/v) Gelzan™, 1 mg/L 2,4-D, and 0.1 mg/L BAP. The cultures were incubated in a standard plant cell culture room under a photoperiod of 16 h light/8 h dark with 45 µmol m^− 2^ s^− 1^ light intensity at a temperature of 25 ± 2 °C. Calli were transferred into fresh medium every two weeks to maintain cell viability.

### Cell suspension culture and growth pattern determination prior to optimization

For cell suspension culture, fourteen-day-old calli were used as inoculum; two grams fresh weight (FW) of calli were cultured in 250-mL Erlenmeyer flasks containing 100 mL of liquid medium at pH 5.8 and supplemented with 1 mg/L 2,4-D and 0.1 mg/L BAP. Cultures were placed on an orbital shaker at 110 rpm under a 16 h light (45 µmol m^− 2^ s^− 1^) /8 h dark photoperiod. Samples were harvested every three days for 30 days. Cells from each flask were harvested and separated from the liquid medium using suction filtration through filter paper (Whatman^®^ No. 1, 70 mm diameter) in a Büchner funnel connected to a vacuum pump. The fresh cells were dried at 45 °C until a constant dry weight (DW) was achieved. Cell morphology was observed using bright-field microscopy (ZEISS Primostar 3, Oberkochen, Baden-Württemberg, Germany). Total betalain content (TBC) was quantified using the methodology outlined in an earlier study^27^. All experiments were performed in triplicate.

### Optimization of culture conditions for enhancing betalain pigment production by *C. argentea* var. *plumosa* using statistical experimental design

Three factors were selected to investigate their effects on betalain production and biomass accumulation in cell suspension culture: BAP (1.33 to 3.11 µM), methyl jasmonate (MeJA) (48.00 to 52.00 µM), and CuSO_4_ (4.40 to 8.40 µM). Response surface methodology (RSM) employing a central composite design (CCD) framework was utilized for statistical evaluation of these variable effects. The coded and actual values of these independent variables are presented in Table 1, where codes A, B, and C represent BAP, MeJA, and CuSO_4_, respectively. The experimental design program determined the lower and upper levels for each variable based on the CCD framework requirements. Each experiment was conducted in triplicate using 250-mL Erlenmeyer flasks containing 100 mL of liquid MS medium (pH 5.8) supplemented with the corresponding factors at the exponential growth phase (on day 9 after cultivation). SCCs underwent a 15-day cultivation period, and samples were collected. Total betalain content (TBC) and cell dry weight (DW) served as the measured response parameters. The experimental design was created using Design-Expert^®^ version 13 software (trial version; Stat-Ease, Inc., Minneapolis, MN, USA; https://www.statease.com). Statistical significance of parameters was assessed through analysis of variance (ANOVA). Subsequently, a validation study was performed under the optimal conditions identified through response surface analysis.

**Table 1:** Codes and actual values of the independent factors for central composite design (CCD) on betalain production from *C. argentea* var. *plumosa*.

Code	Factor	Unit	Level				
			-1.31	-1	0	+ 1	+ 1.31
A	BAP	µM	0.72	1.33	2.22	3.11	3.71
B	MeJA	µM	46.64	48.00	50.00	52.00	53.36
C	CuSO_4_	µM	3.04	4.40	6.40	8.40	9.76

### Growth pattern and betalain production assessment under optimized conditions

Growth patterns and betalain accumulation were examined after obtaining the optimal conditions for all elicitors. Cell suspension cultures were established according to the methodology described in the cell suspension culture and growth pattern determination section prior to optimization. Cultivation was carried out in 250-mL Erlenmeyer flasks containing 100 mL of liquid medium (pH 5.8) supplemented with 1 mg/L 2,4-D and 0.1 mg/L BAP. Elicitors were introduced at the exponential growth phase (day 9 after cultivation), after which the cultures were incubated on an orbital shaker operating at 110 rpm under alternating 16 h light (45 µmol m^− 2^ s^− 1^) and 8 h dark conditions. Sample collection occurred at three-day intervals over a 30-day period. Suspension-cultured cell harvest, dry weight determination, cell morphology analysis, and total betalain content quantification were performed as previously described. Suspension cultures without elicitor addition (supplemented with 1 mL sterile distilled water instead of BAP, MeJA, and CuSO_4_) served as controls. TBC was assessed using the technique described in a previous investigation^27^. All experimental procedures were replicated three times.

### Betalain extract preparation and quantification

Red inflorescence and SCC extracts from *C. argentea* var. *plumosa* were obtained following the methodology outlined in a previous study^27^. In summary, inflorescence and callus powder at 5% (w/v) concentration underwent extraction with distilled water, followed by incubation under agitation at 150 rpm at 50 °C for one hour. The resulting supernatant was subjected to filtration and subsequent lyophilization using a freeze dryer (Christ, Osterode, Germany). The dried betalain powder (5 mg) was reconstituted in 1 mL of distilled water. This reconstituted solution underwent filtration through a 0.2-µm membrane filter prior to analytical procedures. A microplate reader (Infinite^®^ 200 PRO, Tecan Trading AG, Männedorf, Switzerland) was used to determine total betalain content from the extracts. Total betalain content (betaxanthin, BX; and betacyanin, BC) was quantified according to our previous work^27^. In brief, extract samples (350 µL at 5 mg/mL concentration) were analyzed at wavelengths of 483 nm and 535 nm, which represent the optimal absorption maxima for BX and BC, respectively.

### Determination of the antioxidant capacity of betalain extract

The antioxidant capacity was evaluated using the 2,2′-azino-bis(3-ethylbenzothiazoline-6-sulfonic acid) (ABTS) free radical scavenging method, based on protocols described by Smeriglio et al.^30^ and Sang A Roon et al.^27^. The ABTS•+ radical solution exhibits a green coloration that diminishes when exposed to antioxidant compounds. The ABTS•+ radical solution was prepared by combining 4.3 mM potassium persulfate (K_2_S_2_O_8_, w/v in water) with 1.8 mM ABTS solution in a 1:5 volume ratio. The resulting mixture was then incubated for 30 minutes at 25 °C in the absence of light. Before conducting the assay, the ABTS•+ radical solution was diluted using distilled water to obtain an absorbance reading of 0.7 ± 0.02 at 734 nm wavelength. The experimental procedure involved adding 10 µL of each sample extract or ascorbic acid as a positive control (5 mg/mL concentration) to 200 µL of the prepared ABTS•+ solution. Following a 6-minute dark incubation period, absorbance measurements were taken at 734 nm using a microplate reader (Infinite^®^ 200 PRO, Tecan Trading AG, Männedorf, Switzerland).

The 2,2-diphenyl-1-picrylhydrazyl (DPPH) assay was performed based on the protocols described by Shalaby and Shanab with slight modifications^31^. Inflorescence and SCC extracts (5 mg each) were reconstituted in 1 mL of distilled water. A methanolic DPPH radical solution at 0.1 mM concentration was prepared. Using a 96-well microplate format, 100 µL of sample extract (or 5 mg/mL ascorbic acid) was mixed with 100 µL of the DPPH reagent. The reaction mixture was maintained in the dark at 25 °C for 30 min, followed by absorbance measurement at 515 nm wavelength using a microplate reader.

The radical scavenging potential of the extracts was assessed through both ABTS and DPPH assays. The radical inhibition percentage, representing the scavenging capacity, was determined according to Eq. (1):

1 $$\:\mathrm{\%}\:\mathrm{S}\mathrm{c}\mathrm{a}\mathrm{v}\mathrm{e}\mathrm{n}\mathrm{g}\mathrm{i}\mathrm{n}\mathrm{g}\:\mathrm{c}\mathrm{a}\mathrm{p}\mathrm{a}\mathrm{c}\mathrm{i}\mathrm{t}\mathrm{y}\:=\:\left[\right(\mathrm{A}\mathrm{c}\mathrm{o}\mathrm{n}\mathrm{t}\mathrm{r}\mathrm{o}\mathrm{l}\:-\:\mathrm{A}\mathrm{s}\mathrm{a}\mathrm{m}\mathrm{p}\mathrm{l}\mathrm{e})/(\mathrm{A}\mathrm{c}\mathrm{o}\mathrm{n}\mathrm{t}\mathrm{r}\mathrm{o}\mathrm{l}\left)\right]\:\times\:\:100$$

In this Equation, A_control_ represents the absorbance of the diluted ABTS•+ or DPPH radical solution in the absence of test samples, while A_sample_ denotes the absorbance of the combined reaction mixture containing both the diluted radical solution (ABTS•+ or DPPH) and the experimental sample.

The ferric reducing antioxidant power (FRAP) assay was used to determine the antioxidant activity of samples, following the method described by Johnson et al.^32^ with slight modifications. Briefly, the FRAP reagent contained 30 mM acetate buffer (pH 3.6), 30 mM ferric chloride, and 10 mM 2,4,6-tri(2-pyridyl)-s-triazine (TPTZ) dissolved in 40 mM HCl at a ratio of 10:1:1 (v/v/v). Sample extracts (10 µL) from SCC and flowers of *C. argentea* var. *plumosa* were pipetted into 96-well plates with 200 µL FRAP reagent. Vitamin C served as a positive control. The plate was incubated at 37 °C for 4 min, and the antioxidant capacity was determined by measuring absorbance at 593 nm using a microplate reader. A standard curve using iron (II) sulfate (Fe^2+^) (0-4000 µM) enabled the quantification of results as µmol Fe^2+^ equivalents per gram dry weight (DW) of the sample.

All antioxidant assays were conducted in triplicate, and values are expressed as mean ± standard deviation, with error bars indicating standard deviation. Statistical significance was determined using analysis of variance (ANOVA) followed by Duncan’s Multiple Range Test (DMRT), with *p* ≤ 0.05 considered statistically significant.

## Results and discussion

### Cell suspension culture and growth pattern determination prior to optimization

**Fig. 1:**
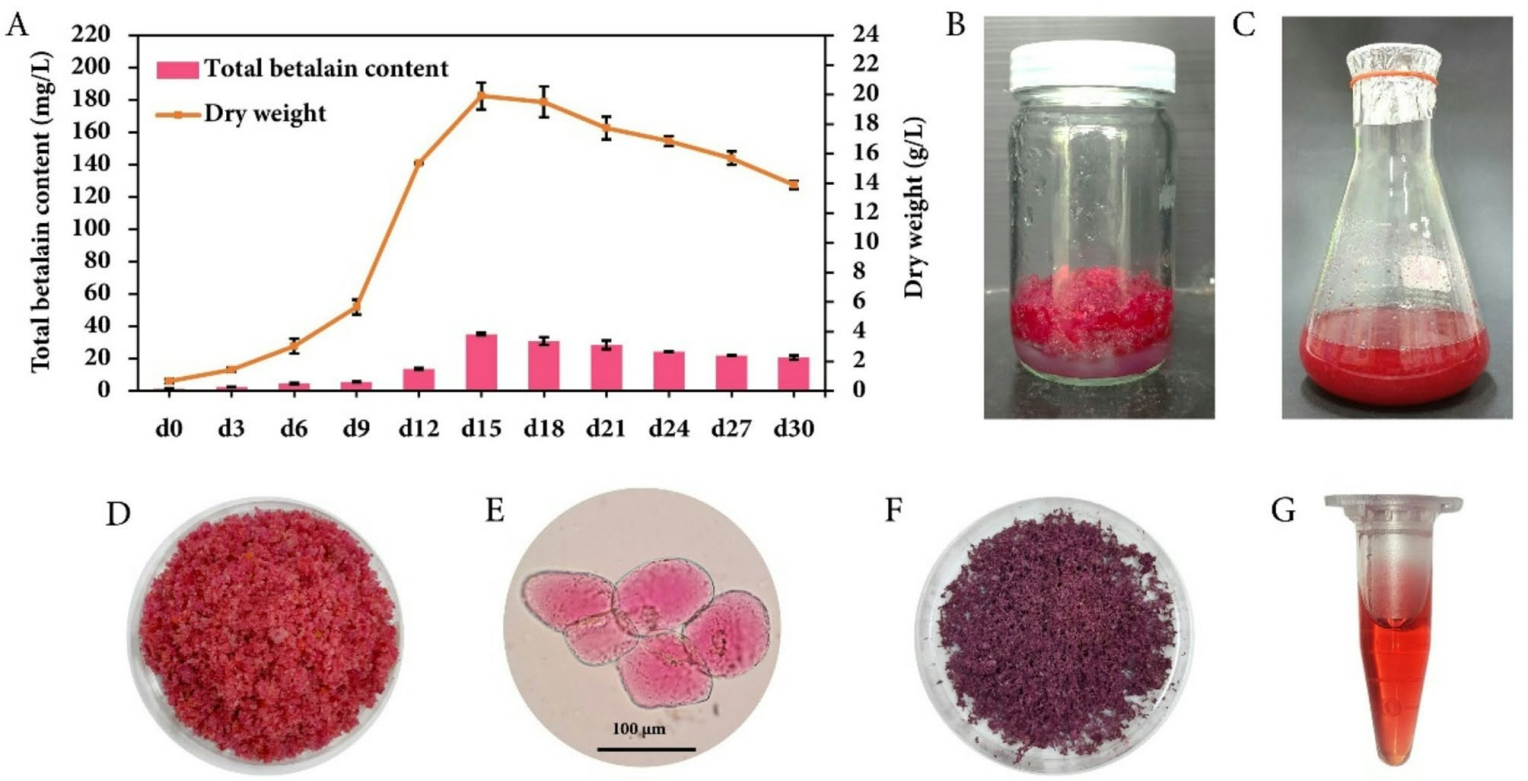
Callus and cell suspension culture of *C. argentea* var. *plumosa*. (**A**) Growth profile with betalain production and cell dry weight; (**B**,** C**) 15-day-old friable callus grown on semi-solid and liquid media; (**D**) 15-day-old suspension culture cells after filtration; (**E**) *C. argentea* var. *plumosa* callus culture cells under microscope; (**F**) dried cells; and (**G**) betalain extract from 15-day-old dried cells.

Betalain production using plant cell culture has been of particular interest due to several notable advantages, including bioactive properties, consistent production independent of seasonal and geographical locations, and the generation of safe pigments^5,15^. In this study, red friable calli with fast-growing characteristics were cultivated in semi-solid MS medium supplemented with 1 mg/L 2,4-D and 0.1 mg/L BAP for establishing cell suspension cultures. The growth pattern of *C. argentea* var. *plumosa* calli in liquid medium under normal conditions supplemented with 3% sucrose (w/v), 1 mg/L 2,4-D and 0.1 mg/L BAP revealed that TBC and DW reached their peak on day 15 with values of 35.61 mg/L (1.95 mg/g DW) and 19.90 g/L, respectively (Fig. 1A). After day 15, the biomass decreased, possibly due to nutrient depletion. The 15-day-old friable calli grown on semi-solid and liquid medium (Fig. 1B and C) showed the same red coloration. This phenomenon indicated that cells grown in different medium types (semi-solid vs. liquid) retained the ability to synthesize and accumulate betalain compounds, indicating a stable cell line. This characteristic suggests that the regulatory mechanisms controlling betalain production in *C. argentea* var. *plumosa* are robust and not significantly affected by physical medium properties, which is crucial for further applications where consistent pigment production is essential.

Suspension-cultured cells (SCCs) on day 15 were separated from liquid medium using vacuum filtration, revealing consistent red-colored cells (Fig. 1D). Bright-field microscopy showed oval and spherical morphologies containing red pigmentation compatible with the presence of betacyanin in vacuoles (Fig. 1E), indicating healthy cell metabolism and proper compartmentalization of betalain compounds. These aspects are essential for maintaining pigment stability due to protection from degradation by enzymes or other cellular components that might be present in the cytoplasm^33^. After fresh SCCs were dried, the color appeared dark red-purple (Fig. 1F), and water extraction of the pigment yielded a red extract (Fig. 1G).

Many studies have investigated betalain production using plant cell cultures in beetroot (*Beta vulgaris*) and other betalain-accumulating plants such as *Amaranthus tricolor*, *Opuntia ficus-indica*, *Hylocereus costaricensis*, *Stenocereus queretaroensis*, *Chenopodium quinoa*, *Pereskia aculeata*, *Celosia argentea* var. *cristata*, and *C. argentea* var. *plumosa*^17^. In the cell suspension cultures of the current investigation, the growth profile and SCC characteristics are consistent with those reported by Mueangnak et al.^29^ and our previous findings regarding the day of highest betalain accumulation, indicating a stable cell line of *C. argentea* var. *plumosa*^27^. However, growth cycles, patterns, and the day of highest betalain accumulation in cell suspension cultures may vary among plant species^10,34^. Notably, similar growth profiles were observed in studies of *C. argentea* var. *plumosa* and *P. aculeata*, whereas a more extended growth period was observed in *H. costaricensis*^10,20,34^.

### Optimization of culture conditions for enhancing betalain pigment production by *C. argentea* var. *plumosa* using statistical experimental design

Successful biomass development and betalain synthesis in suspension culture systems depend on the precise management of various essential elements, including carbon source selection, plant growth regulator types and concentrations, precursor compound availability, and elicitor strategies^17^. The optimal conditions and concentrations for these variables show substantial differences between plant species and cell line types. As a result, thorough optimization of suspension culture parameters is necessary to achieve maximum betalain production efficiency and quantity. Table 1 presents the codes and actual values of the critical factors examined in the experimental design: BAP concentrations (1.33 to 3.11 µM), MeJA concentrations (48.00 to 52.00 µM), and CuSO_4_ concentrations (4.40 to 8.40 µM). The experimental design matrix was evaluated with total betalain content (mg/g) serving as the response variable. Observed TBC values across 20 experimental runs ranged from 1.87 to 7.42 mg/g DW, with predicted values spanning 1.86 to 7.27 mg/g DW (Table 2). A quadratic polynomial regression model and second-order polynomial equation (Eq. 2) were developed to predict TBC (mg/g) based on the culture variables: BAP concentration (A), MeJA concentration (B), and CuSO_4_ concentration (C). The resulting prediction equation is presented as follows:

**Table 2:** The CCD matrix for betalain production using *C. argentea* var. *plumosa*.

Std	Run	BAP (µM)	MeJA (µM)	CuSO_4_(µM)	Total betalain content (mg/g DW)	
					Predicted	Observed
19	1	2.22	50.00	6.40	7.27	7.38
12	2	2.22	53.36	6.40	2.82	2.91
16	3	2.22	50.00	6.40	7.27	7.26
20	4	2.22	50.00	6.40	7.27	7.28
9	5	0.72	50.00	6.40	2.32	2.68
1	6	1.33	48.00	4.40	2.53	2.16
3	7	1.33	52.00	4.40	2.46	2.13
6	8	3.11	48.00	8.40	3.08	3.17
2	9	3.11	48.00	4.40	2.21	1.97
17	10	2.22	50.00	6.40	7.27	7.42
5	11	1.33	48.00	8.40	2.59	2.49
4	12	3.11	52.00	4.40	3.45	3.31
8	13	3.11	52.00	8.40	3.65	3.78
18	14	2.22	50.00	6.40	7.27	6.87
13	15	2.22	50.00	3.04	3.30	3.83
7	16	1.33	52.00	8.40	1.86	1.87
15	17	2.22	50.00	6.40	7.27	7.34
10	18	3.71	50.00	6.40	3.56	3.55
14	19	2.22	50.00	9.76	3.52	3.34
11	20	2.22	46.64	6.40	2.39	2.65

2 $$\begin{aligned} TBC\:\left(\frac{mg}{g}\right)\hspace{0.17em}&=7.27\hspace{0.17em}+\hspace{0.17em}0.3695A\hspace{0.17em}+\hspace{0.17em}0.1272B\hspace{0.17em}+\hspace{0.17em}0.0663C\hspace{0.17em}+\hspace{0.17em}0.3267AB\:\\&+0.02004\mathrm{A}\mathrm{C}\:\--\:0.1653\mathrm{B}\mathrm{C}\:\--\:1.53\mathrm{A}^2\--1.65\mathrm{B}^2\:\--\:1.36\mathrm{C}^2 \end{aligned}$$

The analysis of variance (ANOVA) demonstrated the model’s significance (*p* = 0.0001) with a non-significant lack of fit (*p* > 0.05). The R-squared (R^2^) and adjusted R-squared values were 0.9878 and 0.9769, respectively, indicating a high level of model reliability and predictive accuracy for betalain production. ANOVA was further employed to assess individual factor effects and their interactions on betalain production in *C. argentea* var. *plumosa* SCCs. Statistical analysis revealed significant effects for the linear term of A, the A×B interaction, and the quadratic terms of A, B, and C (Table 3). These findings confirm the substantial influence of BAP, MeJA, and CuSO_4_ on betalain biosynthesis in *C. argentea* var. *plumosa* suspension cultures.

**Table 3:** Analysis of variance (ANOVA) and result of regression analysis of the CCD on betalain production using *C. argentea* var. *plumosa*.

Source	Sum of Squares	df	Mean Square	F-Value	*p*-Value	Note
Model	86.83	9	9.65	90.49	< 0.0001	significant
A-BAP	1.86	1	1.86	17.49	0.0019	
B-MeJA	0.2209	1	0.2209	2.07	0.1806	
C-CuSO_4_	0.0600	1	0.0600	0.5631	0.4703	
AB	0.8536	1	0.8536	8.01	0.0179	
AC	0.3213	1	0.3213	3.01	0.1132	
BC	0.2185	1	0.2185	2.05	0.1828	
A²	33.71	1	33.71	316.13	< 0.0001	
B²	39.15	1	39.15	367.22	< 0.0001	
C²	26.78	1	26.78	251.20	< 0.0001	
Residual	1.07	10	0.1066			
Lack of Fit	0.8662	5	0.1732	4.33	0.0668	not significant
Pure Error	0.2000	5	0.0400			
Cor Total	87.90	19				
C. V. %	7.82					
R-Squared (R^2^)	0.9878					
Adj R-squared	0.9769					

**Fig. 2:**
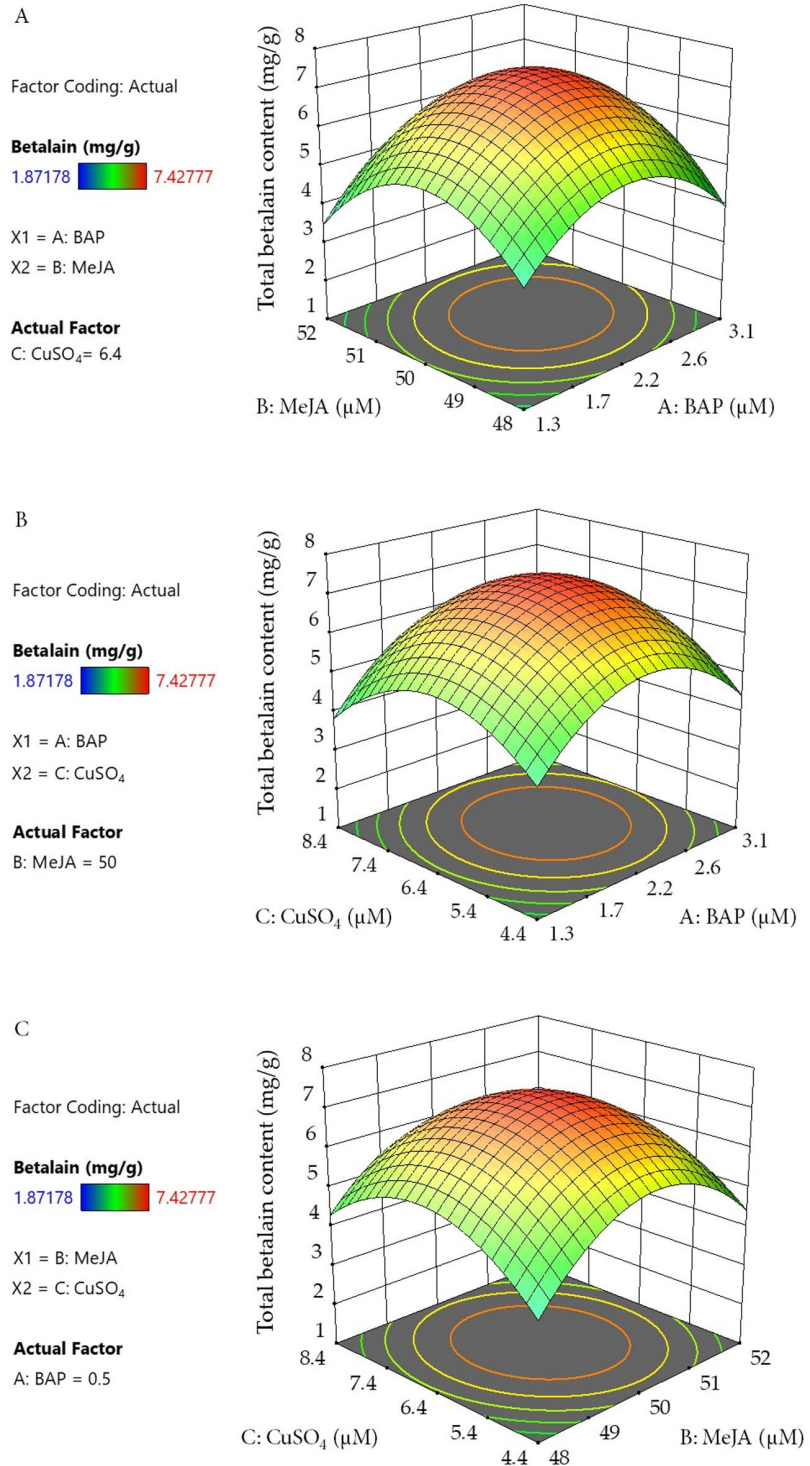
Contour plots and 3-D response surfaces showing the effects and interactions of three factors on betalain production from cell suspension culture of *C. argentea* var. *plumosa*: (**A**) effects of MeJA and BAP concentrations; (**B**) effects of CuSO_4_ and BAP concentrations; and (**C**) effects of CuSO_4_ and MeJA concentrations.

The response surface methodology generated three-dimensional plots and contour maps that illustrate variable interactions in betalain production from *C. argentea* var. *plumosa* SCCs using the developed model and CCD data (Fig. 2). These visualizations demonstrate that elevated concentrations of BAP, MeJA, and CuSO_4_ generally enhance betalain production. However, betalain levels decline when BAP and MeJA concentrations surpass their respective center points of 2.22 µM and 50.00 µM (Fig. 2A). With MeJA maintained at its center point, optimal betalain production occurred at 2.22 µM BAP and 6.40 µM CuSO_4_, corresponding to their center point values (Fig. 2B). Comparable trends were observed when BAP was fixed at its center point (Fig. 2C), confirming the beneficial effects of these parameters within optimal ranges. The integrated analysis of contour plots, response surfaces, and ANOVA results identified BAP, MeJA, and CuSO_4_ as the main factors impacting betalain production in *C. argentea* var. *plumosa* cell suspension cultures. These results offer important guidance for culture condition optimization to enhance betalain production in this system.

Previous research from our laboratory revealed that BAP exerted a substantially beneficial effect on betalain biosynthesis, whereby elevated BAP levels up to an optimal point resulted in enhanced betalain production. Conversely, tyrosine, which serves as a biosynthetic precursor for betalain formation, did not significantly influence betalain synthesis in *C. argentea* var. *plumosa* cell suspension cultures^27^. Multiple investigations have reported the application of BAP at relatively low concentrations (generally below 8.88 µM) to enhance betalain accumulation in both callus and cell suspension cultures of *Chenopodium quinoa*, *A. tricolor*, *C. argentea* var. *cristata*, and *C. argentea* var. *plumosa*^8,11,20,35^. In agreement with these reports, our investigation showed that BAP concentration optimization had a favorable impact on betalain synthesis in *C. argentea* var. *plumosa* cell suspension cultures. It should be emphasized, however, that elevated BAP levels can result in cellular mortality, as documented in *Daucus carota* and *Arabidopsis thaliana* plant cells^36^.

The application of diverse elicitor compounds represents one of the most successful approaches for enhancing the biotechnological synthesis of plant secondary metabolites, including betalains^37^. Various investigations have examined the utilization of both biotic and abiotic elicitors across different concentration ranges to enhance betalain synthesis in hairy root systems, callus cultures, and cell suspension cultures of betalain-producing species, such as *B. vulgaris*, *C. argentea* var. *cristata*, *Bougainvillea* spp., *S. queretaroensis*, *Portulaca* sp., and *H. costaricensis*^19,21,23,28,38,39^. Nevertheless, research examining the application of external abiotic elicitors in *C. argentea* var. *plumosa* cell suspension cultures remains limited to a single investigation. This research demonstrated that MeJA (50.00 µM) and CuSO_4_ (6.40 µM) substantially enhanced betalain synthesis, achieving concentrations of 3.32 and 4.99 mg/g DW, respectively^29^. Our findings align with these observations, as the optimal concentrations of abiotic elicitors within the hormonal and chemical abiotic elicitor category (BAP, MeJA, and CuSO_4_) were comparable and demonstrated beneficial effects on betalain synthesis in *C. argentea* var. *plumosa* cell suspension cultures.

### Growth pattern and betalain production assessment under optimized conditions

Optimal conditions for betalain production in *C. aregentea* var. *plumosa* cell suspension cultures were determined through statistical experimental design utilizing three-factor quadratic polynomial equations and CCD experiments. These optimized parameters were subsequently validated through confirmatory studies and benchmarked against standard cultivation conditions. The growth profile of *C. argentea* var. *plumosa* suspension cultures and total betalain content (TBC) were assessed every three days throughout a 30-day cultivation period following optimization.

**Fig. 3:**
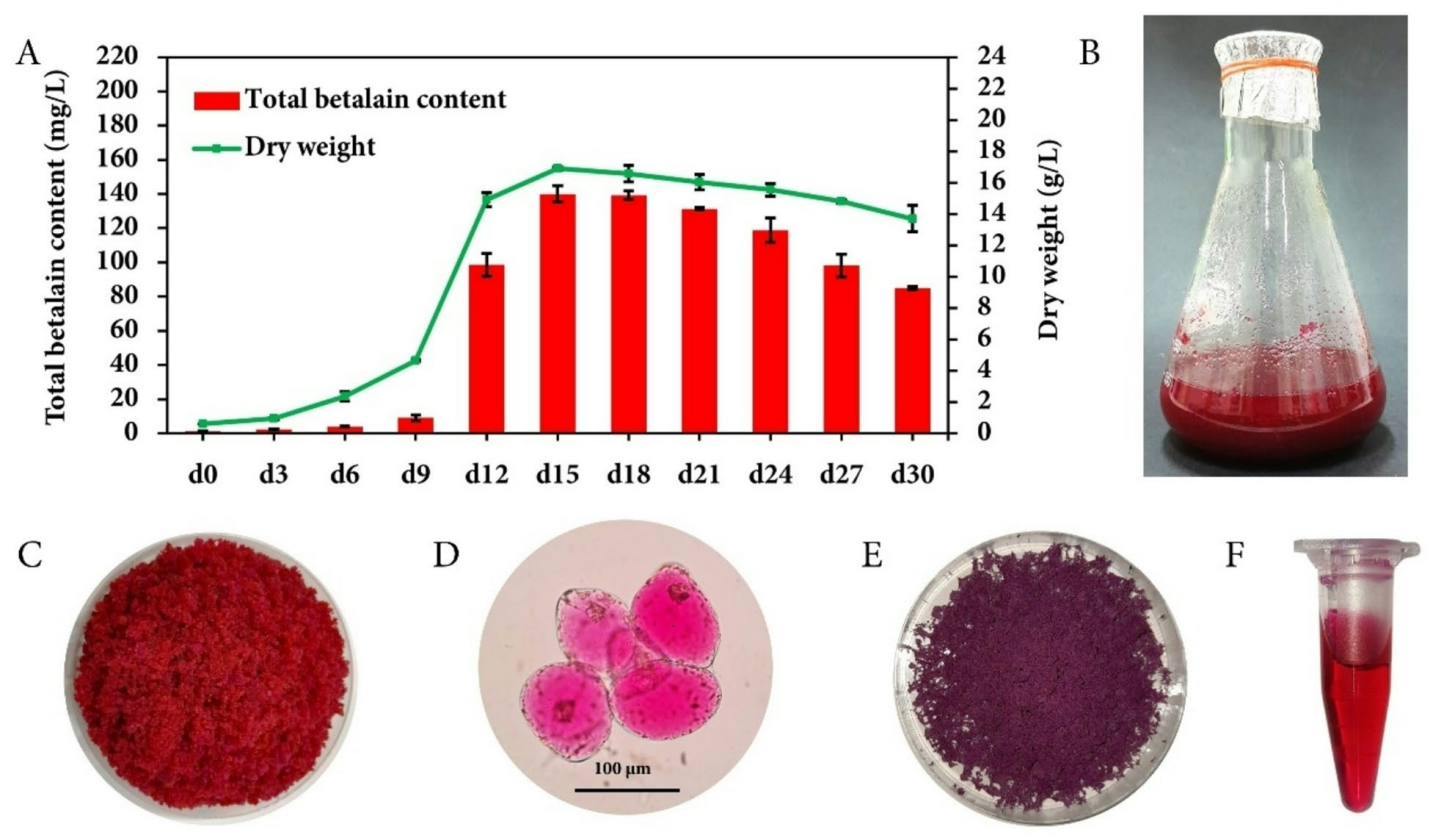
Growth profile and betalain production from cell suspension culture of *C. argentea* var. *plumosa* in liquid medium under optimum conditions. (**A**) Total betalain content and dry weight of cell suspension culture; (**B**) 15-day-old cell suspension culture; (**C**) fresh friable cells after filtration; (**D**) bright-field morphology of 15-day-old cells; (**E**) 15-day-old dried cells after water evaporation; and (**F**) crude betalain extract from dried *C. argentea* var. *plumosa* suspension cells after optimization.

The validated medium formulation from RSM consisted of 2.28 µM BAP, 49.97 µM MeJA, and 6.71 µM CuSO_4_, which was supplemented to the suspension culture during day 9, corresponding to the exponential growth phase. Cell proliferation proceeded continuously, achieving peak biomass accumulation on day 15. Under these optimized conditions, the cultures yielded maximum TBC and DW values of 139.99 mg/L (7.54 mg/g DW) and 16.90 g/L, respectively (Fig. 3A).

After reaching the maximum, both betalain synthesis and biomass accumulation declined. This reduction might be attributed to nutrient depletion and the onset of cell death. Filtration of the 15-day suspension culture (Fig. 3B) revealed distinctly crimson-pigmented cells (Fig. 3C). Morphological analysis was conducted on fresh cell samples. Microscopic evaluation showed cellular morphology consistent with pre-optimization cultures, displaying characteristic oval and spherical configurations, but with intensified red pigmentation (Fig. 3D) that was notably more pronounced than observed in unoptimized conditions. Aqueous extraction of day-15 dried biomass (Fig. 3E) produced a concentrated deep-red pigment extract, suggesting high betacyanin content, which is desirable for both research applications and potential commercial use as natural colorants (Fig. 3F).

The current study demonstrated a stable betalain-accumulating cell line with predictable growth patterns and betalain accumulation profiles, providing a foundation for optimizing production parameters. After optimization, the highest TBC of 139.99 mg/L (7.54 mg/g DW) was observed on day 15 after culture initiation. This represents approximately 3.93-fold higher betalain production compared to pre-optimization conditions (35.61 mg/L / 1.95 mg/g DW). The present study achieved higher betalain content compared to Mueangnak et al.^29^ who evaluated individual elicitors separately. Their study showed that CuSO_4_ at 6.40 µM produced the highest betalain content of 4.99 mg/g DW, which was lower than the values obtained in the present investigation. These results are consistent with findings by Lakhotia et al.^38^, who reported that CuSO_4_ addition, specifically at 20 µM, significantly increased betacyanin and betaxanthin accumulation in callus cultures of *Bougainvillea* cv. Bhabha. Similarly, Trejo-Tapia et al.^40^ demonstrated that CuSO_4_ at 5 µM enhanced betalain production in suspension cultures of *B. vulgaris*.

The enhanced betalain production observed with CuSO_4_ treatment in suspension cultures of *C. argentea* var. *plumosa* may be attributed to increased activity of enzymes involved in the betalain biosynthesis pathway. CuSO_4_ can serve as a cofactor for key enzymes, particularly tyrosinase, which is crucial for the early stages of betalain biosynthesis−specifically the initial conversion of tyrosine to L-DOPA, a precursor for betalain production^41^. However, contrasting results were reported by Warhade and Badere^21^, who found that CuSO_4_ concentrations exceeding 6 µM dramatically reduced the synthesis of betacyanin, including amaranthin, betanin, and betalamic acid, in cell suspension cultures of *C. argentea* var. *cristata*. Interestingly, they observed preferential accumulation of betaxanthin when 6.40 µM CuSO_4_ was added to the culture medium.

Methyl jasmonate (MeJA) has been recognized as an important signaling compound in the elicitation process in callus and cell suspension cultures, resulting in the promotion of various secondary metabolites, including betalains^29,42^. Based on the contour plots and 3-D response surface analysis, the present study identified 49.97 µM as the optimal MeJA concentration for maximizing betalain yield in *C. argentea* var. *plumosa* cell suspension cultures, achieving 7.54 mg/g DW. This finding correlates with previous investigations conducted by Mueangnak et al.^29^, who examined MeJA concentrations ranging from 0 to 200 µM in *C. argentea* var. *plumosa* cultures. It was demonstrated that the maximum betalain content (3.32 mg/g DW) was achieved in cell suspension cultures when 50 µM MeJA was added to the culture medium. Notably, MeJA concentrations exceeding 100–200 µM led to decreased betalain synthesis^29^. Additional research by Lakhotia et al.^38^ revealed that minimal MeJA concentrations (0.5 µM) substantially increased betacyanin and betaxanthin levels in *Bougainvillea* spp. callus cultures. Comparable enhancement was observed in *Portulaca* sp. cv. ‘Jewel’ cell suspensions, where 0.1 µM MeJA treatment resulted in a 2.6-fold betacyanin increase^39^.

Research studies suggest that MeJA can promote secondary metabolite accumulation in plant cells through the mechanism of activating transcription of various genes involved in their biosynthesis. This activation enhances the expression of genes encoding enzymes in betalain biosynthesis pathways, leading to increased betaxanthin and betacyanin production in callus cultures of *Bougainvillea* and cell suspension cultures of *C. argentea* var. *plumosa*^29,38^. Supporting this mechanism, Milech et al.^43^ demonstrated that 100 µM MeJA treatment significantly upregulated key betalain biosynthetic genes, notably *cytochrome P450 CYP76AD1* (*CYP76*) and *DOPA 4*,*5-dioxygenase* (*DODA*), in *Alternanthera sessilis*. Transcriptomic analysis of *A. tricolor* leaves confirmed the existence and functionality of betalain metabolic networks, providing detailed gene identification and expression profiling^44^. Based on these findings, future research should focus on gene expression analysis within betalain biosynthetic pathways in *C. argentea* var. *plumosa* cell cultures following optimization protocols incorporating BAP, MeJA, and CuSO_4_.

### Determination of the antioxidant capacity of betalain extract

**Fig. 4:**
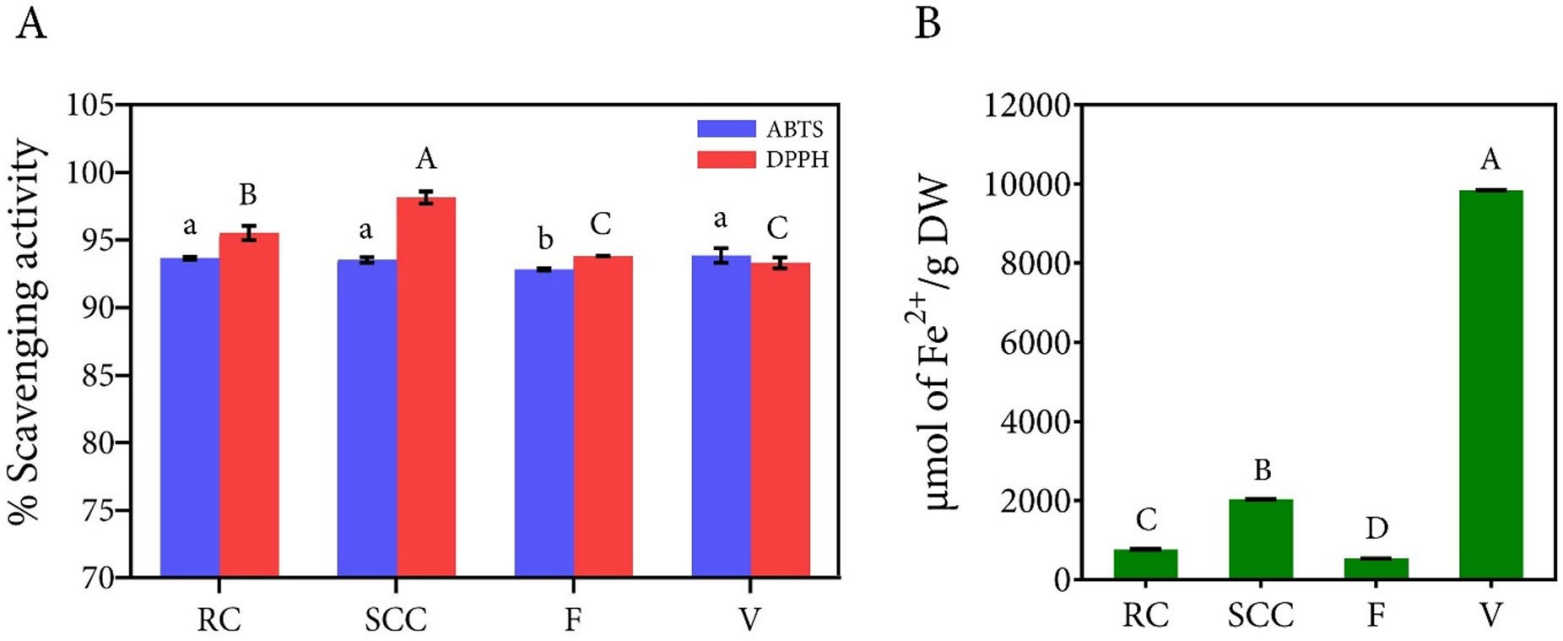
Antioxidant capacity of SCC extracts from *C. argentea* var. *plumosa* determined by (**A**) ABTS and DPPH assays and (**B**) FRAP assay. Data are presented as means ± SD of triplicate determinations. Different capital and lowercase letters within each assay indicate significant differences between samples. All statistical analyses were performed using Duncan’s Multiple Range Test (DMRT) at *p* ≤ 0.05. RC (red callus; control), SCC extract from cells cultured under unoptimized conditions; SCC, SCC extract from cells cultured under optimized conditions; F (flower), inflorescence extract; and V (vitamin C), ascorbic acid.

The antioxidant capacity of extracts from inflorescence (F), unoptimized culture (RC), and optimized cultures (SCC) was evaluated using 2,2′-azino-bis(3-ethylbenzothiazoline-6-sulfonic acid) (ABTS), 2,2-diphenyl-1-picrylhydrazyl (DPPH), and ferric reducing antioxidant power (FRAP) assays with vitamin C (V) serving as a positive control for antioxidant compound. The ABTS scavenging activity of the optimized culture extract (93.52%) was comparable to the control (93.65%), with both values exceeding that of the inflorescence extract (92.82%) (Fig. 4A). Although all samples exhibited slightly lower ABTS scavenging activity than vitamin C, the difference was not statistically significant for the extract from the optimized cultures. In the DPPH assay, the highest antioxidant capacity was observed in the optimized SCC extract (98.14%), followed by the extract from unoptimized cultures (95.51%). The inflorescence extract and vitamin C exhibited antioxidant capacities of 93.80% and 93.32%, respectively, both of which were lower than the values obtained from the optimized culture extracts.

The FRAP assay results demonstrated that the optimized SCC achieved a FRAP value of 2,036 µmol Fe^2+^/g DW, representing a substantial improvement over both the control (769.75 µmol Fe^2+^/g DW) and inflorescence extract (538.31 µmol Fe^2+^/g DW) (Fig. 4B). While all plant extracts exhibited lower antioxidant capacity compared to vitamin C (9,841.67 µmol Fe^2+^/g DW), the optimized SCC showed a 2.6-fold increase in antioxidant activity compared to the control and a 3.8-fold increase relative to the inflorescence extract. The FRAP value obtained for vitamin C in this study was consistent with that reported by Benzie and Strain^45^.

The optimized *C. argentea* var. *plumosa* cell cultures showed higher antioxidant activity than both unoptimized cultures and natural inflorescence extracts, with the most significant differences observed in the DPPH and FRAP assays. This enhanced antioxidant activity can be attributed to the increased betalain content achieved through optimization procedures, as betalains are well-documented for their potent antioxidant properties^4,5,14,46^. The optimized SCC extract achieved 98.14% DPPH scavenging activity and a FRAP value of 2,036 µmol Fe^2+^/g DW, indicating that our optimization conditions successfully enhanced both betalain production and antioxidant capacity in the culture system. For DPPH assay, this value exceeded even vitamin C (93.32%), a standard antioxidant reference compound, indicates the potential of these optimized culture extract as a source of natural antioxidants for commercial applications.

The ABTS results showed similar scavenging activities between optimized cultures (93.52%) and controls (93.65%), confirming that our optimization maintained antioxidant performance while increasing betalain yield. This aspect is particularly important for industrial applications^2,3^. The lower antioxidant capacity observed in inflorescence extracts compared to SCC extracts may be attributed to several factors, such as seasonal and geographical variations, which may cause inconsistency in pigment production^15,16^. Cell suspension cultures, being maintained under controlled conditions, can provide more consistent antioxidant profiles, making them advantageous for standardized production.

The optimized SCC performed differently across the antioxidant assays, suggesting that multiple methods are required when evaluating antioxidant capacity. Although the extract showed strong activity in DPPH and ABTS assays, the lower FRAP values compared to vitamin C reflect differences in assay mechanisms^47,48^. The FRAP assay measures ferric reducing power via electron transfer mechanisms at low pH, which may not detect all antioxidant compounds in complex plant extracts^45^. DPPH and ABTS assays operate through different mechanisms, involving hydrogen atom transfer (HAT) and single electron transfer (SET). The superior performance in DPPH and ABTS suggests that the bioactive compounds in *C. argentea* var. *plumosa* extracts are more effective at radical scavenging, which may be more relevant in biological systems with multiple oxidative stress pathways.

These results align with previous studies demonstrating the correlation between betalain content and antioxidant activity in various betalain-accumulating plant species such as *C. argentea* var. *plumosa*, *Amaranthus* spp., *B. vulgaris*, *H. costaricensis*, and *O. ficus-indica*^9,10,29,49,50^. Our optimized culture conditions for *C. argentea* var. *plumosa* improved both betalain production and antioxidant capacity, suggesting this system has good potential and could serve as natural alternatives to synthetic antioxidants for safe use in food preservation, cosmetics, and dietary supplements.

## Conclusion

This study developed an optimized cell suspension culture system for enhanced betalain production from *Celosia argentea* var. *plumosa*. Response surface methodology optimization identified optimal culture conditions of 2.28 µM BAP, 49.97 µM MeJA, and 6.71 µM CuSO_4_, which increased betalain production by 3.93-fold compared to standard conditions, achieving total betalain content of 139.99 mg/L (7.54 mg/g DW) and 35.61 mg/L (1.95 mg/g DW), respectively. Extracts from optimized cultures showed improved antioxidant properties, with 98.14% DPPH scavenging activity that exceeded both unoptimized cultures and vitamin C controls. This culture system using BAP, MeJA, and CuSO_4_ offers potential for consistent betalain production with year-round availability, regardless of geographic location. The enhanced bioactive properties suggest possible applications in food, pharmaceutical, and cosmetic industries requiring natural colorants with functional benefits.

Although the findings are promising, several limitations should be considered. The optimization was carried out only at the laboratory scale, which may not reflect betalain production performance in larger production systems, where several factors could be affected. Additionally, the optimization considered only three types of elicitors (BAP, MeJA, and CuSO_4_), while other factors, including additional elicitor types or environmental parameters, may further enhance production efficiency. Furthermore, the research focused primarily on total betalain content without detailed analysis of individual betalain compounds, which limits our understanding of the qualitative changes in betalain composition under optimized conditions and their potential applications in different industries. Therefore, further studies are needed to evaluate the scalability of this system in bioreactor conditions and assess its commercial viability for large-scale production. Additionally, investigating the metabolite profiles and the molecular mechanisms underlying the synergistic effects of BAP, MeJA, and CuSO_4_ on betalain biosynthesis could inform the development of more efficient production strategies.

### Submission declaration and verification

Submission of an article implies that the work described has not been published previously in any form.

## Data Availability

The datasets generated during and/or analyzed during the current study are available from the corresponding author on reasonable request.
